# High-Sensitivity C-Reactive Protein and Magnetic Resonance Imaging in Occult Giant Cell Arteritis

**DOI:** 10.7759/cureus.9530

**Published:** 2020-08-03

**Authors:** Katrina A Mears, Doron Feinsilber

**Affiliations:** 1 Ophthalmology, Retina Consultants of Southwest Florida/National Ophthalmic Research Institute, Fort Myers, USA; 2 Hematology/Oncology, Medical College of Wisconsin/Froedtert Cancer Center, Milwaukee, USA

**Keywords:** giant cell arteritis, temporal artertitis, tocilizumab, cell reactive protein, erythrocyte sedimentation rate, optic neuropathy

## Abstract

Giant cell arteritis (GCA) can be an elusive diagnosis and is particularly challenging to monitor during the course of treatment when traditional acute phase reactants are all normal, leaving no empirical means of monitoring. Our study aims to explore the use of more sensitive acute phase reactants and imaging in the initial evaluation and monitoring of GCA. We report the case of an 84-year-old in whom the traditional acute phase reactants were normal but who had perineuritis on imaging and whose high-sensitivity C-reactive protein (CRP) levels were elevated. In cases where the traditional measure of inflammatory activity is normal, it may be necessary to consider additional markers.

## Introduction

We present a case of occult giant cell arteritis (GCA) that highlights the importance of having a low threshold for considering the diagnosis of GCA in elderly patients with sudden or subacute visual loss, especially when both eyes are affected simultaneously. The occult disease is defined as the ocular involvement of GCA without any systemic symptoms and signs of GCA. Although our patient did experience visual loss, there were no other classic symptoms or elevation of routine acute phase reactants in him. Our study aims to explore the use of more sensitive acute phase reactants and imaging in the initial evaluation and monitoring of GCA.

## Case presentation

An 84-year-old Caucasian male presented to his primary ophthalmologist three weeks after undergoing an uncomplicated right cataract extraction. He complained of sudden onset of blurred vision in his right eye upon waking up and was found to have a visual acuity of light perception in the right eye and 20/60 in the left eye with optic disc swelling and disc hemorrhages in both his right and left eyes. This marked a change from his best-corrected visual acuity at one week following cataract extraction, which had been 20/40 in his right eye and 20/25 in his left eye. Fluorescein angiography was done within a week of symptom onset, which showed no abnormalities. Specifically, there was no delay in the choroidal filling.

**Figure 1:**
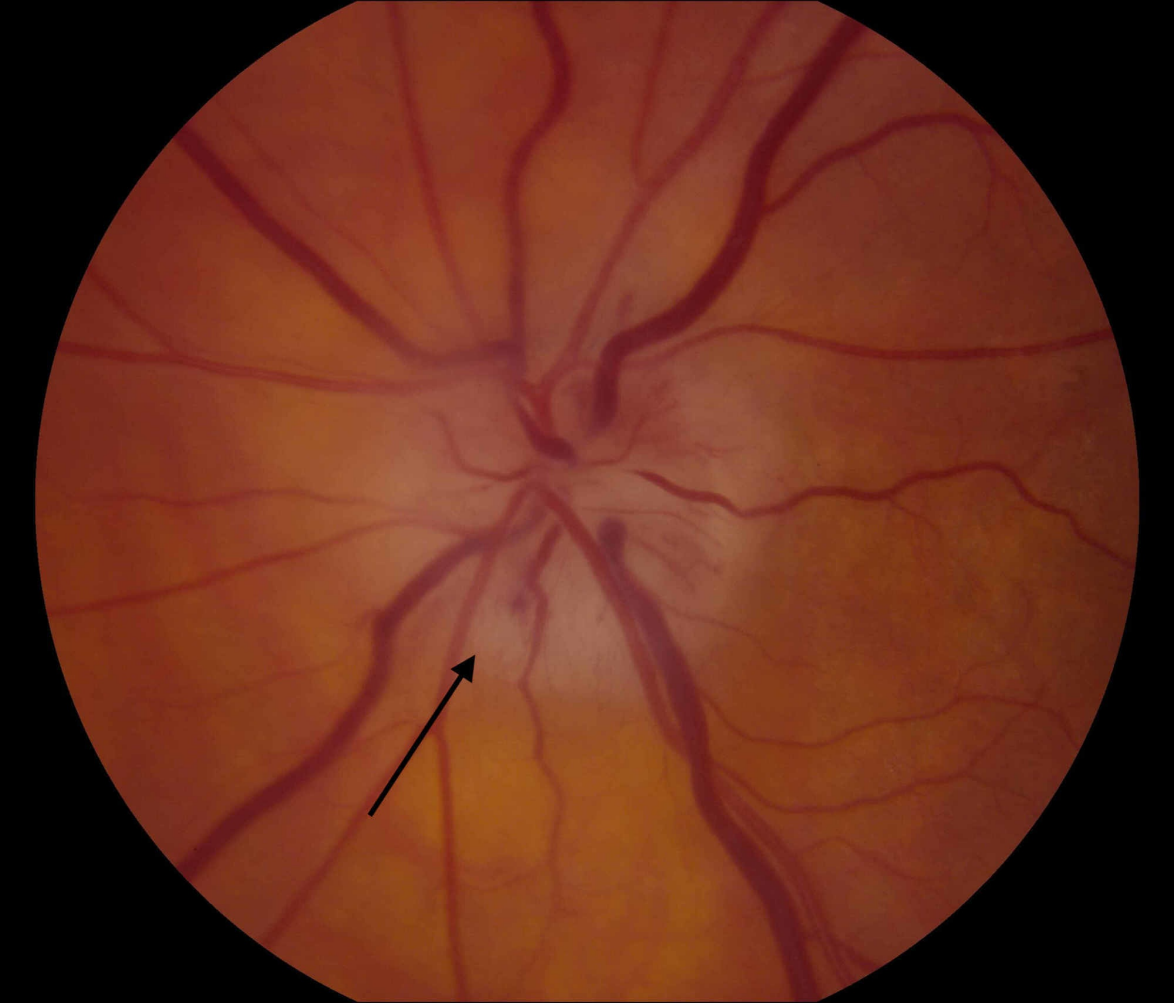
Right optic nerve showing chalky pallor with swollen disc (arrow)

**Figure 2:**
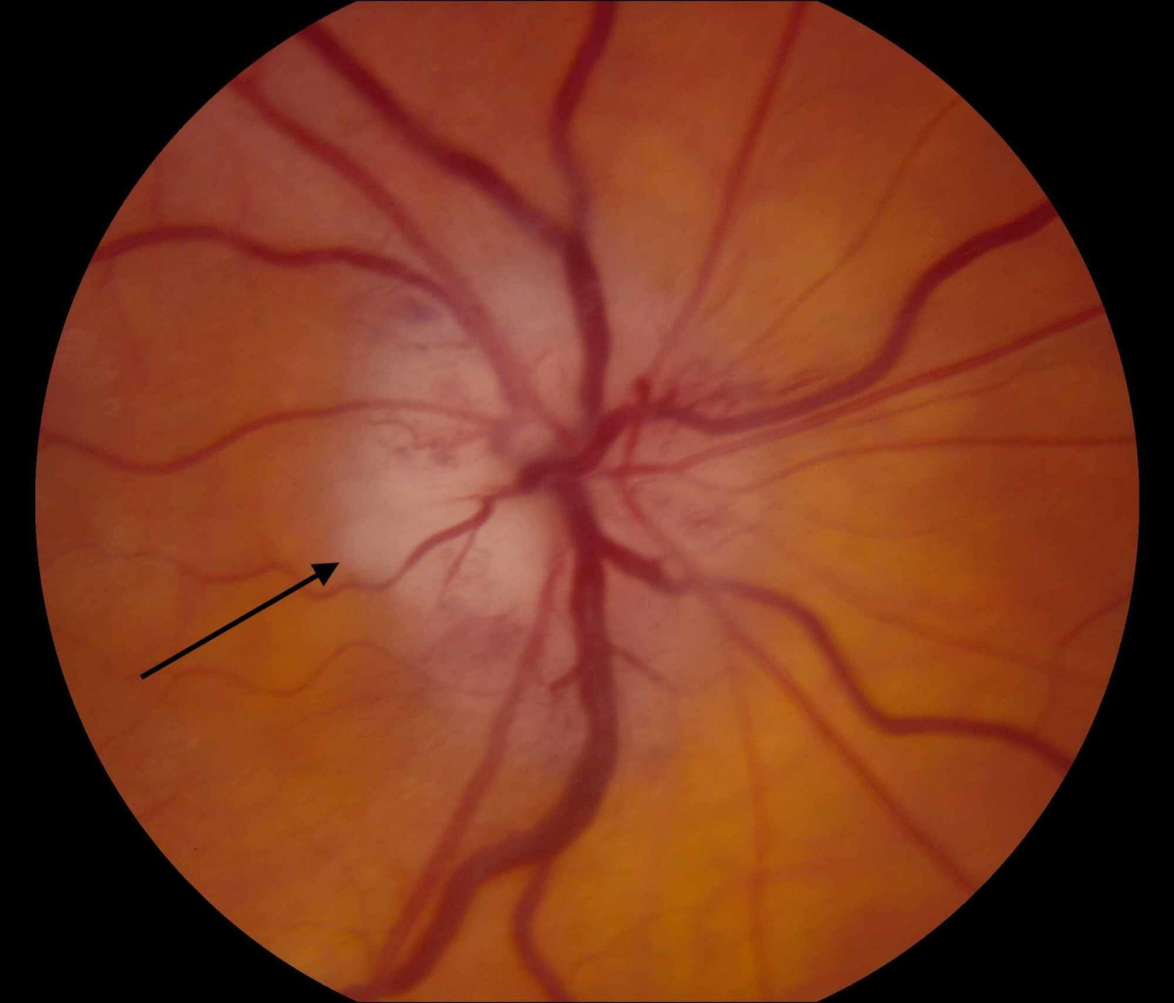
Left optic nerve showing chalky pallor and swollen optic nerve with arrow showing marked chalky pallor nasally

Upon examination at our institution, he denied jaw claudication, headache, scalp tenderness, fever, weight loss, rheumatological complaints, or any other medical problems. Visual fields were unobtainable from the right eye due to poor visual acuity and were full in the left eye to confrontation. A right relative afferent pupillary defect was present, and the left pupil appeared to react normally. He was pseudophakic OU and had normal intraocular pressures of 10 mmHg in each eye. He was found to have bilateral disc swelling, mildly attenuated vessels, and normal periphery (Figures 1, 2).

**Figure 3:**
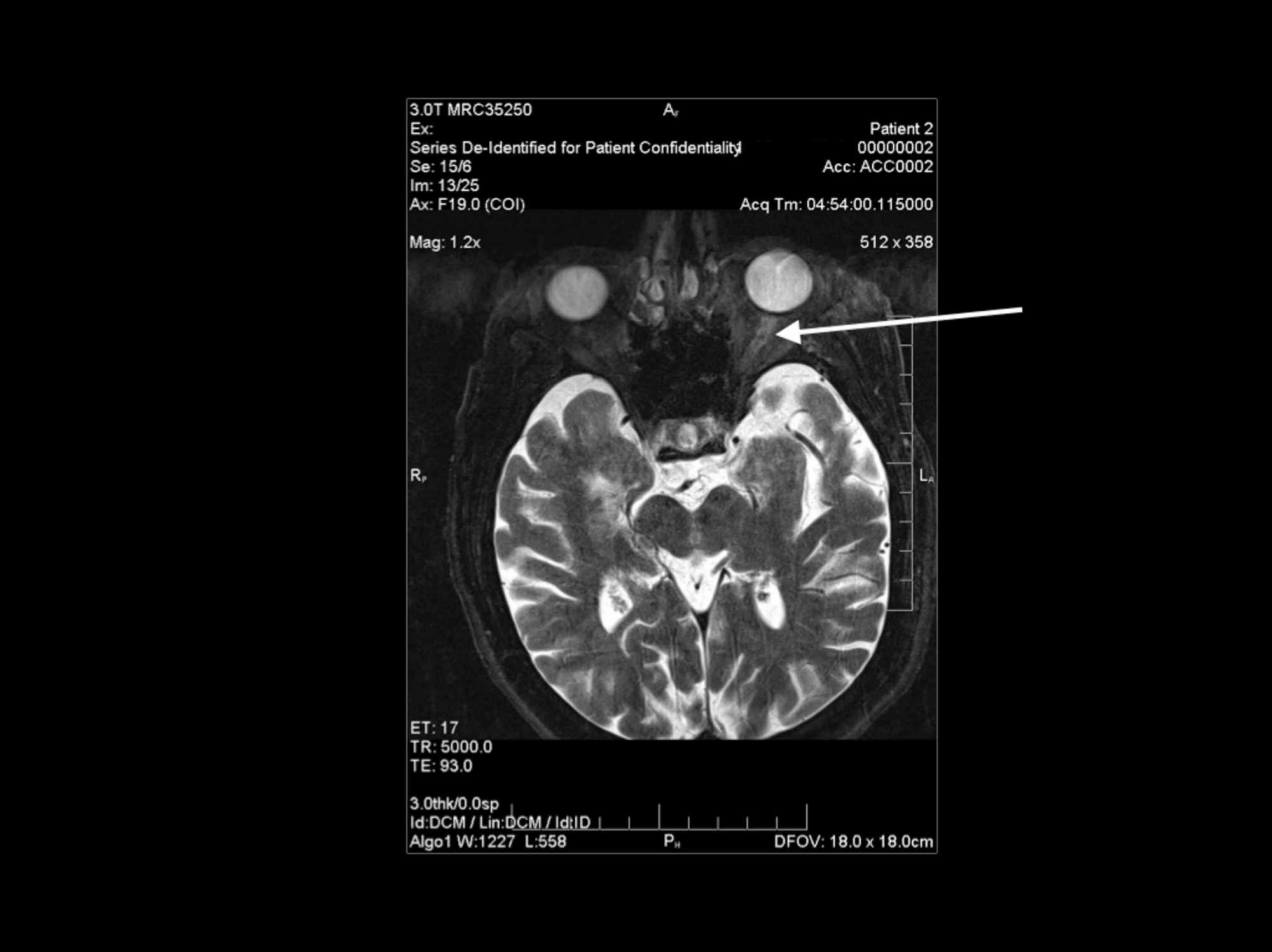
MRI showing perineural enhancement of the left optic nerve sheath (arrow) MRI: magnetic resonance imaging

Laboratory results revealed an erythrocyte sedimentation rate (ESR) of 14 mm/hour, C-reactive protein (CRP) of 2.1 mg/L, platelet count of 134,000/microliter, hematocrit of 48.4%, and hemoglobin of 16.6 g/dL. However, high-sensitivity CRP was elevated at 22.10 mg/L. The patient was admitted for further evaluation to the neurology service and, because of concern for an intracranial mass or atypical optic neuritis, MRI of the brain and orbits was obtained. This showed enhancement of the left optic nerve sheath (Figure 3).

**Figure 4:**
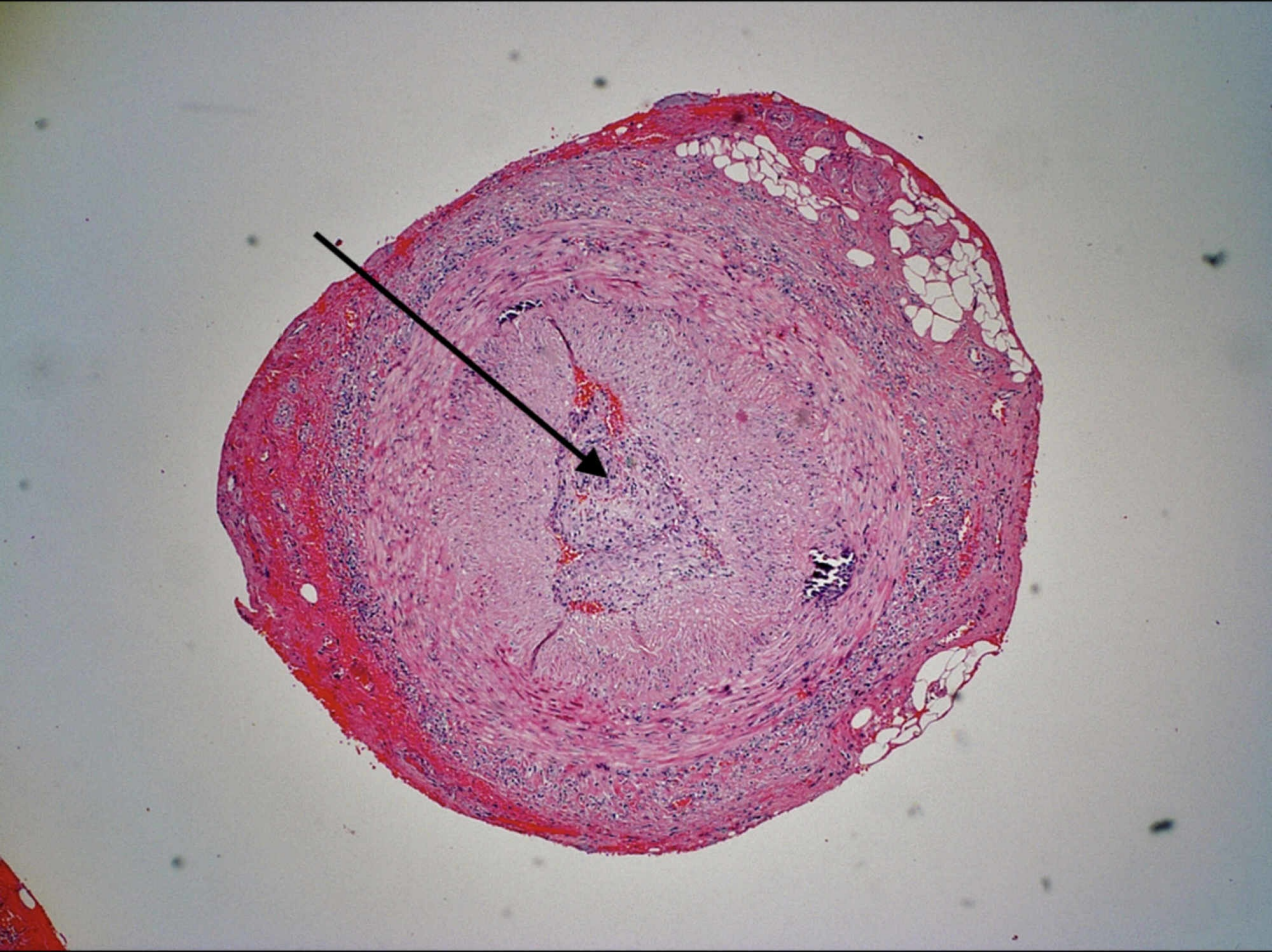
Pathology slide showing the temporal artery lumen mostly obliterated by subintimal and mural inflammation (arrow)

The neurology service proceeded with a more focussed workup for optic neuritis, seeking out infectious as well as vasculitic causes. A lumbar puncture was performed and tested for neuromyelitis optica, syphilis, and lupus, which came back negative. He was commenced on 250 mg intravenous methylprednisone qid and 81 mg of aspirin. A bilateral temporal artery biopsy was performed the next day and was positive on each side. This demonstrated panarteritis consisting of lymphocytes and macrophages without granuloma formation. Additionally, intimal thickening and fragmentation of the internal elastic lamina was demonstrated (Figure 4).

Currently, four months after his initial presentation, the patient still has macular vision in the left eye. The swollen optic discs and hemorrhages have resolved, and he has optic disc pallor in both eyes as expected. He is currently taking 60 mg of oral prednisone each day, and this is being slowly tapered based on close monitoring of his clinical exam and high-sensitivity CRP value.

## Discussion

The causes of bilateral disc swelling with visual loss are wide-ranging and include intracranial mass lesions, inflammatory, infectious and autoimmune optic neuropathies, ischemic optic neuropathies, and dysthyroid optic neuropathy [1]. In addition, disorders of chronic increased intracranial pressure also need to be considered. However, the rapidity and severity of visual loss and the presence of bilateral optic disc edema in our patient raised the specter of temporal arteritis.

Although our patient did experience visual loss, there were no other classic symptoms or elevation of routine acute phase reactants in him. One multi-center retrospective trial has found CRP to have a higher sensitivity than ESR (61 versus 71%) and a specificity of 57% for ESR versus 68% for CRP [2]. To date, there are no studies in the literature that have examined the role of high-sensitivity CRP in the diagnosis of GCA. This may be attributed to the fact its utility mainly lies in cardiovascular disease and in the prediction of cardiovascular morbidities and mortality [3]. Its role is of particular interest in atypical cases such as this one, where the routine acute phase reactants remain normal and a more sensitive marker is required. Delayed filling in the choroidal circulation was absent in either eye on the original fluorescein angiogram done within one day of visual loss. Delayed filling in the choroidal circulation has been described as a diagnostic feature of GCA and implies ischemia affecting the tributaries of the posterior ciliary circulation, which are branches of the ophthalmic artery. The MRI showed enhancement of the left optic nerve sheaths, suggestive of a perineuritis (Figure 2). Perineuritis has a multitude of etiologies including sarcoidosis, GCA, granulomatosis with polyangiitis, neurosyphilis, tuberculosis, and idiopathic causes. Recent studies have reported its rare association with GCA [4].

Corticosteroids or a steroid-sparing agent is usually required in the treatment of GCA for at least one to two years following the diagnosis; however, even after years of clinical stability and normal laboratory results, re-exacerbations of the disease have been reported. Although there is no established standard of care for GCA presenting with visual loss, our treatment plan included three days of intravenous methylprednisone 250 mg qid followed by 1 mg/kg day of oral prednisone with a slow taper over an extended period based on clinical signs and symptoms and high-sensitivity CRP levels, since all other acute phase reactants, including routine CRP, remained normal. Of note were reports describing a genetically determined blunted cytokine response [CD4 Th17 cell types producing interleukin-17 (IL-17)] in certain patients with GCA [5]. This may explain why acute phase reactants such as ESR and CRP are not elevated in some patients. Recent reports show the beneficial use of aspirin in the treatment of patients with GCA, especially in preventing subsequent stroke; its mechanism is postulated to be either the inhibition of interferon-gamma production or the inhibition of thrombus formation [6]. However, the salutary effect of aspirin use in patients with GCA is controversial. In recurrent or resistant GCA, methotrexate or other immunosuppressives such as azathioprine or leflunomide may be used as adjuncts [7-9]. Newer biological agents such as tocilizumab, an IL-6 receptor inhibitor, have also been used and has shown promise at maintaining remission at 52 weeks in a phase-3 randomized controlled clinical trial [10]. Abatacept, a cytotoxic T lymphocyte-associated protein 4 fusion protein, has shown a significant increase in relapse-free survival at 12 months [11]. Ustekinumab, an anti-IL-12 and -23 monoclonal antibody, functions by inhibiting both the Th1 and Th17 pathways simultaneously, and has shown promising results in some studies [12,13].

## Conclusions

Maintaining a low threshold for the diagnosis of GCA in patients over the age of 65 years with vision loss is crucial in identifying cases of occult GCA. Additionally, the use of high-sensitivity CRP test is worth considering in cases of suspected occult GCA. Temporal artery biopsy still remains the gold standard for the diagnosis of GCA.
